# THE PERSISTENCE OF COMBAT-RELATED PTSD AMONG MALE VIETNAM WAR VETERANS

**DOI:** 10.1093/geroni/igae098.0941

**Published:** 2024-12-31

**Authors:** Avron Spiro, Anica Pless Kaiser, Brian Smith, Jeanne Stellman, Steven Stellman

**Affiliations:** VA Boston Healthcare System, Boston, Massachusetts, United States; VA Boston Medical Center, Jamaica Plain, Massachusetts, United States; Women’s Health Sciences Division, National Center for PTSD, Boston, Massachusetts, United States; Columbia University, New York, New York, United States; Columbia University, New York, New York, United States

## Abstract

The US involvement in the Vietnam War ended 50 years ago, yet many who served in that conflict, especially those who served in combat, continue to carry the impact of their military service into their later lives. For many those impacts have been positive, and for others the negative impacts have dissipated over time. However, for a sizable minority the long-term repercussions of combat deployment during their youth continue to reverberate across the lifespan. We present data on 507 male Vietnam combat veterans who were American Legion members. They were surveyed by mail in 1984, 1998, and 2020. We examined (1) patterns of PTSD over time, (2) the relation of combat exposure to these patterns, and (3) the impact of these PTSD patterns on health in later life. First, 56% of the sample did not meet PTSD criteria at any time; 25% evidenced fluctuating subthreshold PTSD, 10% had prior but not current PTSD, and 9% met criteria for current PTSD in 2020. Second, the strong dose-response relationship between combat exposure and PTSD symptoms first reported in 1988 has persisted over nearly a half-century. Finally, we found that the pattern of persistent PTSD in later life was associated with greater depression, anxiety, and disability, and with worse health functioning, and more mental health utilization. These observations extend to veterans with subthreshold PTSD as well. Across their lifetime since returning from Vietnam, a substantial number of US veterans continue to experience PTSD and its negative impact on their health and well-being.

